# Immunomodulatory, Anti-Inflammatory, and Anti-Cancer Properties of Ginseng: A Pharmacological Update

**DOI:** 10.3390/molecules28093863

**Published:** 2023-05-03

**Authors:** Jose Antonio Valdés-González, Marta Sánchez, Ignacio Moratilla-Rivera, Irene Iglesias, María Pilar Gómez-Serranillos

**Affiliations:** Departamento de Farmacología, Farmacognosia y Botánica, Facultad de Farmacia, Universidad Complutense de Madrid, Plaza Ramón y Cajal s/n, Ciudad Universitaria, 28040 Madrid, Spain

**Keywords:** ginsenosides, ginseng, immunomodulation, anti-inflammatory, anti-cancer, saponins, polysaccharides

## Abstract

Ginseng, a medicinal plant of the genus *Panax*, boasts a rich historical record of usage that dates back to the Paleolithic period. This botanical is extensively acknowledged and consumed in Eastern countries for its therapeutic properties, and, in Western countries, it is becoming increasingly popular as a remedy for fatigue and asthenia. This review provides an update on current research pertaining to ginseng and its isolated compounds, namely, ginsenosides and polysaccharides. The primary focus is on three crucial pharmacological activities, namely, immunomodulation, anti-inflammatory, and anti-cancer effects. The review encompasses studies on both isolated compounds and various ginseng extracts obtained from the root, leaves, and berries.

## 1. Introduction

Ginseng, a highly valued herbal medicine, has been used for over 5000 years, predominantly in Far East countries. Emperor Shen-Nung of China was the first to identify ginseng, along with several other herbs, as having medicinal properties, and developed what is considered the first pharmacopoeia in history [1]. Ginseng is the common name for the root of various species of the genus *Panax (P.)*, belonging to the Araliacea family. The genus name, *Panax*, is derived from the ancient Greek “Panákeia” (Πανάκεια), which translates to “all healing” or “cure of all diseases.” Similarly, the term “ginseng” originates from the Chinese “jen-shen”, which means “plant-man”, possibly referencing the human form of the root [2,3]. The Greek term “panacea”, which shares the same etymological roots as “panax”, also implies that the ginseng root is a genuine and authentic panacea.

Archaeological research indicates that the ginseng root has been used as a medicinal plant since the Paleolithic period, dating back approximately 60,000 years ago [4]. The use of ginseng as a medicinal plant has been predominantly concentrated in China and other Asian countries, where it grows naturally. In East Asian countries, ginseng has been used as an adaptogenic drug since its discovery in the Manchurian mountains, more than 5000 years ago [5]. The Shen-Nung Benchau Jing treatise is considered the oldest pharmacopoeia in the world, and the use of ginseng is already mentioned in it, revealing that its pharmacological potential was already known [6].

**Figure 1:**
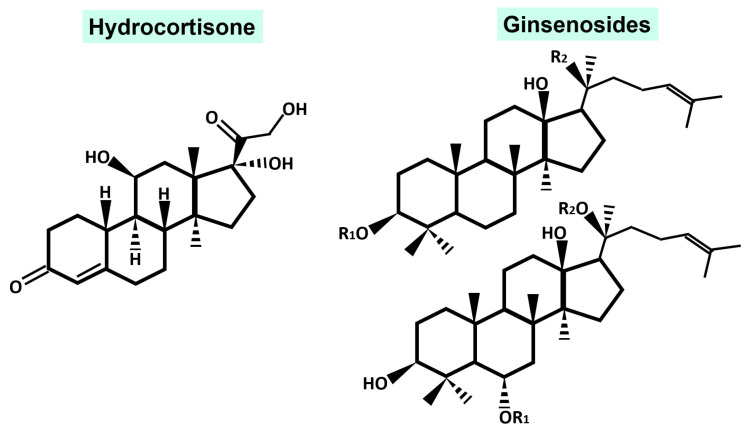
Comparison of the molecular structure of a human endogenous anti-inflammatory drug (hydrocortisone) with ginsenosides.

Different species of ginseng have been used for centuries, due to their anti-inflammatory properties, which are closely associated with their ability to modulate the immune system. The chemical structure of the main active compounds, ginsenosides, partly explains this activity [7]. The chemical structure of ginsenosides is very similar to the structure of hydrocortisone, the most significant endogenous anti-inflammatory molecule [7,8,9] as shown in Figure 1. Both hydrocortisone and most ginsenosides consist of three aromatic rings that form a phenanthrene molecule linked to a cyclopentane to create a skeleton of cyclopentane-perhydrophenantrene. But this structure, by itself, does not justify the pharmacological activity of ginsenosides. This activity depends on the substituents and unsaturations of each ginsenoside, as we will see later. Thus, the anti-inflammatory activity varies depending on the ginsenoside studied, according to its chemical structure. Many ginsenosides have shown immunomodulatory properties, as well as polysaccharides associated with these ginsenosides. The primary immunomodulatory activity is due to these polysaccharides. One of the mechanisms by which ginseng exerts its immunomodulatory activity is by increasing nitric oxide (NO) production and a nonspecific stimulation of lymphocyte proliferation [10]. A recent study has investigated in depth the immunomodulatory capacity of the polysaccharides of North American ginseng (*P. quinquefolius*), using the crude extract from the root, as well as partially purified and completely isolated polysaccharides. However, the limited information available about the structure of these polysaccharides poses a challenge in determining the relationship between structure and activity, although some mechanisms have been proposed [11]. Furthermore, ginseng compounds, such as ginsenosides, polysaccharides, and alkaloids, can exert cancer-protective activity by inhibiting cell proliferation through the regulation of cell cycles and apoptosis.

Currently, there is a growing trend in the use of herbal medicinal products for the treatment of various diseases [12]. This increase can be attributed to various factors, including societal perception of these treatments as being healthier than conventional drugs. Additionally, there is a common misconception that herbal medicines present fewer adverse effects, and therefore are perceived as more “natural” treatments [13]. Furthermore, research on natural products is of great importance at present. Many plant families, genera, and species remain unexplored, presenting a potential source of molecules that could serve as appropriate treatment for diseases such as cancer, Alzheimer’s, or Parkinson’s [4,12]. Consumption habits of ginseng vary greatly from country to country, but, globally, it is valued at over two billion dollars. Ginseng is often consumed together with other natural products, such as royal jelly, vitamins, and minerals, but it can also be consumed alone [5]. All abbreviations used are listed in the Abbreviation.

## 2. Phytochemical Characteristic

The genus Panax consists of eleven species, including P. ginseng, P. notoginseng, P. quinquefolius, P. pseudoginseng, P. trifolius, P. zingiberensis, P. stipuleanatus, P. japonicus, P. japonicus var. angustifolius, P. japonicus var. major, and P. japonicus var. bipinnatifidus [14]. Morphologically, they are characterized by herbaceous perennial plants with palmate leaves with serrated margins. Generally, ginseng species reach 60–80 cm in height, and produce flowers ranging from white to purple, these flowers appear in an umbel at the apex. In addition, these species have a complex root system, comprising of a short rhizome and many tuberous roots [15]. While the most used species for their pharmacological properties are P. ginseng, P. notoginseng, and P. quinquefolius, all these species contain bioactive compounds of pharmacological interest [16]. 

**Figure 2:**
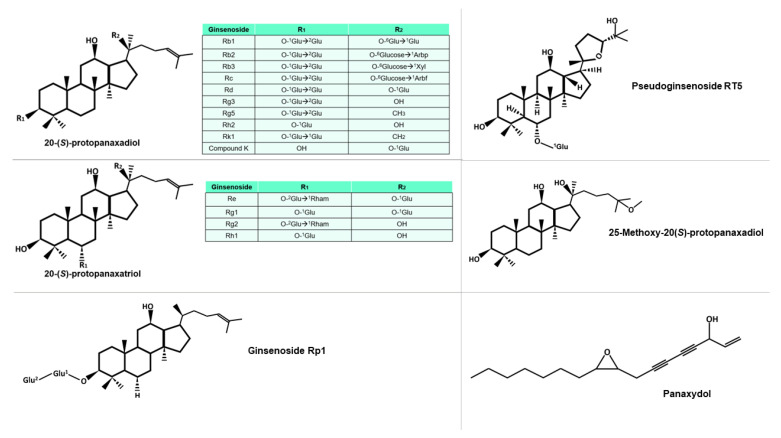
Diversity of protopanaxadiol and protopanaxtriol-type ginsenosides, characterized by the glucidic substituents they possess, including Glu (glucose), Arbp (arabinose in pyranose form), Xyl (xylose), Arbf (arabinose in furanose form), and Rham (rhamnose). The superscript notation of the sugar indicates the specific carbon involved in the bond. Other molecules mentioned in this study are also included in the figure.

Ginsenosides, the primary bioactive compounds found in ginseng, can be classified into five groups, based on their distinct chemical structures: protopanaxadiol, protopanaxatriol, ocotillo, oleanolic acid, and C-17 side-chain type [17]. The structural diversity of ginsenosides is attributed to the presence of various radicals and sugar moieties. Furthermore, the primary structure of some ginsenosides can be modified to produce secondary ginsenosides through natural microbiota [18], or by various processes during the preparation of the phytotherapeutic product, such as heating or drying. Some of these molecules can be seen in Figure 2. Additionally, the composition of each ginseng preparation can be influenced by factors such as growth temperature, altitude, and weather conditions, in the different places of cultivation [19]. Although ginsenosides are the most extensively studied molecules among the components of ginseng, other bioactive compounds, such as polysaccharides, glycolipoproteins, and alkaloids, also exhibit important pharmacological activities. 

The structure/activity relationship of ginsenosides depends on each molecule, as well as on each specific bioactivity. In any case, for most studies, the molecular mechanisms are not deeply elucidated, but focus on the consequences of these mechanisms. For example, we will see that oleanane-type ginsenosides possess greater immunomodulatory activity than dammarane-type ginsenosides, or that the anti-cancer activity of ginsenosides increases the lower the number of sugar residues in the molecule. However, the precise molecular mechanism underlying these biological consequences remains, in most cases, unelucidated.

Chromatographic methods, such as High-Performance Liquid Chromatography (HPLC), Thin-Layer Chromatography/High-Performance Liquid Chromatography (TLC/HPLC), Ultra-High-Performance Liquid Chromatography (UHPLC), and Two-Dimensional High-Performance Liquid Chromatography (HPLC 2D), are commonly employed, in combination with spectroscopic analysis, to determine the molecular composition of different species of the *Panax* genus. Using these techniques, researchers have discovered up to 623 types of ginsenosides in the ethanol extract of the most used species, of which 437 are potentially novel ginsenosides [20]. Additionally, 945 ginsenosides, and 662 potentially novel ginsenosides, have been identified from *P. notoginseng* leaves [21,22].

## 3. Immunomodulatory Activity

The immune system plays a vital role in defending the body against external threats. In humans, it is comprised of two distinct components, namely, the innate immune system and the adaptive immune system. Each system is equipped with unique cells and molecules to perform its specific functions. Macrophages and Natural Killer Cells (NK cells) are well-known cells of the innate immune system, whereas T lymphocytes and B lymphocytes are good examples of the adaptive immune system. To achieve successful immunity against pathogens, effective communication between these two systems is essential. In this context, the present discussion focuses on how various molecules found in ginseng can influence the immune system, and a summary is provided in Table 1.

Ginseng has been extensively studied for its bioactivity in modulating the immune system, a property also observed in other medicinal plants [23]. Initially, it was suggested that ginseng polysaccharides were responsible for this immunomodulatory effect [9]. However, subsequent investigations have revealed the involvement of various ginsenosides, including RT5, Rh2, oleanolic acid β-D-glucopyranosyl ester [24], Rh1 [25], Rg3 [26], and Rb1 [27], in triggering this biological response.

*P. ginseng* extracts have demonstrated noteworthy pharmacological bioactivities, including the improvement of macrophages’ phagocytic activity and enhanced production of NO [28,29]. Furthermore, ginseng extracts have been shown to boost interleukin 12 (IL-12) release [30]. Scaglione et al. [31] conducted a study at two levels, in vivo and with humans, demonstrating interesting results that can be immediately applied in clinical treatments, such as improvements in chemotaxis, phagocytosis index, and phagocytosis fraction. Additionally, different ginseng extracts have been observed to regulate various cytokines and molecules involved in immunomodulation, including IL-1α, IL-1β, IL-6, tumor necrosis factor α (TNF-α), NO, inducible nitric oxide synthase (iNOS), or cyclooxygenase-2 (COX-2) [32,33]. Therefore, ginseng extracts possess bioactive compounds that can regulate the immune system, resulting in a normalization of its functioning. The observed effect may be attributed to the presence of specific compounds or a synergistic activity among them, which may produce a more potent effect.

The earliest investigations into the immunomodulatory effects of ginseng attributed its bioactivity to its polysaccharide content [34,35,36,37,38,39]. The underlying mechanisms of action have been partially elucidated, with TNF-α being the primary stimulus, and other molecules, such as NO, IL-6, and IL-1β, also being stimulated [40]. In addition to the increased production of these immune-associated molecules, ginseng extracts have been shown to induce a Th1 immune response and activate pathways, such as nuclear factor κB (NF-κB), mitogen-activated protein kinases (MAPK), and phosphatidylinositol 3-kinase (PI3K) [14].

The primary distinction between ginseng extract responses depends on the mode of extraction and presentation, as well as the molecular weight of bioactive compounds. Notably, a conflicting effect was observed in vivo by Azike et al., who reported that, while the aqueous extract of ginseng polysaccharides increased TNF-α and NO levels, it also inhibited the physiological rise of these proinflammatory mediators induced by lipopolysaccharide (LPS). TNF-α levels were determined by Enzyme-Linked InmunoSorbent Assay (ELISA), and NO production was estimated by assessing nitrite accumulation with the Griess reagent. Treated animals displayed a reduction of around 50% in NO production, compared to non-treated animals [37]. This effect can be explained by considering that ginseng exerts an immunomodulatory action, instead of a stimulatory or inhibitory effect.

Ginsan, an acidic polysaccharide, has been found to possess unique abilities in stimulating the production of inflammatory mediators, such as NO, by upregulating iNOS, which sets it apart from other ginseng extracts [41]. However, ginsan also exhibits other bioactivities related to the immune response, such as the induction of T helper type 1 (Th1) cells and the release of cytokines produced by macrophages [42]. In addition, ginsan has multiple immunomodulatory effects on dendritic cells, including enhancing the expression of cluster of differentiation 86 (CD86) on their surface and increasing the levels of IL-12 and TNF-α secreted by them [43].

Recent studies have challenged the previous notion that polysaccharides are solely responsible for the immunomodulatory bioactivity of ginseng. Instead, ginsenosides and ginsenoside-like molecules have been shown to play an important role through distinct, and sometimes overlapping, mechanisms [44,45]. In vitro studies have demonstrated that ginsenoside RT5 and ginsenoside Rh2 increase IL-2 production. Interestingly, oleanane-type triterpenoids have been found to exhibit stronger immunomodulatory effects than those of the dammarane type [24]. Additionally, ginsenoside Rh2 has been found to increase the number of T cells in mice with melanoma, linking its immunomodulatory effect with anti-cancer properties [46]. Animal studies involving ginsenoside Rb2 have also revealed a higher survival rate and reduced tumor size [46]. 

In vitro studies have demonstrated the immunomodulatory activity of ginsenoside Rh1. Its action is complementary to that of polysaccharides, which regulate proinflammatory mediators, such as TNF-α or IL-6. Ginsenoside Rh1 reduces the expression of various proinflammatory mediators, including TNF-α, IL-1β, IL-6, IL-17, and NO. Additionally, it suppresses enzymes such as matrix metalloproteinase 1 (MMP-1), iNOS, and COX-2 [47,48]. In obese mice, Rh1 has been shown to exhibit immunomodulatory properties, by suppressing proinflammatory cytokines such as TNF-α, IL-1β, and IL-6 [49]. In hairless female mice, oral administration of Rh1 resulted in reduced IL-6 and immunoglobulin E (IgE) levels in peripheral blood analysis [50]. These findings suggest that Rh1 has some level of bioavailability through this route of administration; however, further research is necessary to clarify this point.

In various studies, other ginsenosides isolated as pure compounds have also demonstrated immunomodulatory properties. For instance, Rg3 has been found to enhance Fc gamma receptor-mediated phagocytosis in macrophages through mechanisms related to the activation of extracellular signal-regulated kinase 1/2 (ERK 1/2) and p38 [25]. Additionally, Rg3 has been shown to regulate cytokines and transcription factors, thereby maintaining homeostasis [51]. Ginsenoside Rg3 has been shown, in a study with patients with non-small cell lung cancer, to improve the immune response against COVID-19 by modulating the immune system against this disease. The same study shows the ability of ginsenoside Rg3 to regulate cell cycle, suggesting a possible application against different types of cancer [52].

Another compound, Rb1, has been found to increase both humoral and cell-mediated immune responses through the induction of antigen-presenting cells, which secrete TNF-α, and T cells, which secrete interferon gamma (IFN-γ) and IL-10. Rb1 also induces the production of immunoglobulins such as IgA, IgG1, and IgG2, and potentiates virus-triggered IFN-γ expression [27]. In another study, Rb1 was found to ameliorate the expression of TNF-α and IL-6 [53].

Rg1 has demonstrated many mechanisms through which it can carry out its activity related to immunomodulation. Studies, both in vivo and in vitro, have shown that Rg1 can activate the nuclear factor E2-related factor 2 (Nrf2) signaling pathway, which generates protection of the liver from toxins and disease [54,55,56]. Furthermore, both ginsenoside Rg1 and ginseng extracts have been shown to improve NK cells activity [57].

One of the main problems in therapy with ginsenosides is the great difficulty to absorb them. In fact, the bioavailability of ginsenosides from the intestinal mucosa is very low, and its transport through intestinal mucosa to blood is energy-dependent and non-saturable [3].

Compound K, which is not a main ginsenoside, but is closely related to them and derived from their biotransformation, is of clinical significance, due to its better bioavailability and numerous immune system activities. It attenuates NF-κB by modulating the protein kinase B (PKB or Akt)-mediated inflammatory gene expression [58], and regulates cytokines and other immune molecules such as IL-1β, IL-6, TNF-α, COX-2, and iNOS [59,60,61]. However, Compound K has also been shown to suppress humoral immune response of Th1 cells and suppresses the expression of matrix metalloproteinases and receptor activator of NF-κB ligand (RANKL) [62]. Additionally, it can inhibit β-arrestin2, hindering the transformation of macrophages from type M1 to type M2 [63].

**Table 1:** Immunomodulatory bioactivity of ginseng extracts and compounds. An increase is represented by (↑), and a decrease is represented by (↓).

Species	Molecular Group	Compound/Extract	Experimental Model	Result	Ref.
*Panax quinquefolius* L.	Polysaccharides	Extract	Wistar rats	↑ Macrophages activity	[34]
	Polysaccharides	Extract	Human peripheral blood mononuclear cells	↑ Pro-inflammatory cytokines	[35]
	Polysaccharides	Extract	Mouse 3T3-L1 preadipocytes	Cytokines regulation	[36]
	Polysaccharides	Extract	Sprague–Dawley rats Murine RAW 264.7 macrophage cell line	Cytokines regulation	[37]
*Panax ginseng* C.A. Meyer	Polysaccharides	Acidic fraction	C57BL/6 mice macrophages	Cytokines regulation	[38]
	Polysaccharides	Acidic fraction	C57BL/6 mice	Enhanced phagocytic effect	[41]
	Polysaccharides	Acidic fraction	C57BL/6 mice dendritic cells	↑ CD86	[43]
	Ginsenosides	Rh1	Murine RAW 264.7 macrophage cell line	Glucocorticoid receptor stimulus	[47]
	Ginsenosides	Rh1	Hartley guinea pigs, SD rats, and ICR mice	↓ NO ↓ PGE_2_	[48]
	Ginsenosides	Rh1	Mouse embryo fibroblasts 3T3-L1 cells	↓ TNF-α ↓ IL-1β ↓ IL-6	[49]
	Ginsenosides	Rh1	Hairless mice	↓ Infiltration of inflammatory cells ↓ IgE levels	[50]
	Ginsenosides	Rb1	EV71 mice model	↑ Cellular immune response ↑ Humoral immune response	[27]
	Ginsenosides	Rg1	C57BL/6 mice C57BL/6 mice hepatocytes	↑ Nrf2 ↑ Detoxifying enzymes	[55]
	Ginsenosides	Rg3	BALB/c mice	Improve immune system	[51]
	Ginsenosides	Rg3	Patients with non-small cell lung cancer	Regulate cell cycle	[52]
	Ginsenosides	Standardized G-115 extract	BALB/c pathogen-free mice	↑TLR4	[40]
	Ginsenosides	Compound K	Murine RAW 264.7 macrophage cell line Human Embryonic Kidney cell line (HEK293 cells)	↓ iNOS ↓ TNF-α	[58]
	Ginsenosides	Compound K	Sprague–Dawley rats Kunming mice	Cytokines regulation	[61]
	Ginsenosides	Compound K	DBA/1 OlaHsd mice	↓ Th1 response (in arthritis)	[62]
	Ginsenosides	Compound K	DBA/1 mice	Alleviates inflammatory response	[63]
	-	Extract	Clinical trial	↑ Chemotaxis	[31]
	-	Extract	Murine RAW 264.7 macrophage cell line BALB/c mice	Cytokines regulation	[28]
	-	Extract	Balb/C mice C57 B1/6J mice C57 B1/6J nu/nu mice	↑ Antibody formation ↑ NK	[29]
	-	Extract	Murine RAW 264.7 macrophage cell line	Cytokines regulation	[32]
	-	Extract	Murine RAW 264.7 macrophage cell line	↑ Immunomodulators	[33]
*Panax ginseng* C.A. Meyer *Eleutherococcus senticosus* Rupr. & Maxim	-	Extract	Mouse J774A.1 macrophages	↑ lL-12	[30]

## 4. Anti-Inflammatory Activity

Inflammation is an integral part of the immune response, and, therefore, the immunomodulatory actions are of considerable significance in this regard [61]. While inflammation serves as a physiological response to protect against pathogenic infections, it poses a risk factor in various human diseases such as neurodegenerative, cardiovascular, autoimmune, respiratory disorders, and even certain cancers. Therefore, controlling or moderating the immune response may prove to be a useful strategy in preventing complications arising from inflammation. The results of recent studies on the anti-inflammatory activity of ginseng are shown in Table 2.

Compound K has been reported to possess anti-inflammatory properties. The mechanisms underlying these bioactivities include the downregulation of inflammatory cytokines such as IL-1β, IL-6, and TNF-α [64,65,66], as well as the modulation of reactive oxygen species (ROS) generation, Mitogen-activated protein kinases (MAPKs), NF-κB, and activator protein 1 (AP-1) [64,65,66,67,68]. 

Apart from specific ginsenosides, ginseng aqueous extracts have also been evaluated for their anti-inflammatory effects. For instance, in rats intoxicated with fipronil, an insecticide that triggers oxidative stress and induces inflammatory responses, *Panax ginseng* aqueous extract normalized the levels of reduced glutathione (GSH) and catalase (CAT) activity [69]. Ginseng aqueous extracts can be divided into two fractions, namely, saponin and non-saponin fractions. Although ginsenosides belong to the saponin fraction, which has been shown to reduce the generation of inflammatory molecules and messenger RNA (mRNA) levels of inflammatory cytokines and enzymes in vitro [70], both fractions have been reported to exert anti-inflammatory effects [71].

In the investigation of ginseng, the root is typically examined. However, studies have shown that ginseng berry extracts possess anti-inflammatory properties in macrophages induced by LPS. Specifically, ginseng berry extract reduces the production of NO and prostaglandin E2 (PGE2), as well as the expression of enzymes such as iNOS or COX-2. Moreover, it diminishes the secretion of cytokines such as IL-1β, IL-6, and TNF-α, and inhibits the translocation of NF-κB by avoiding the phosphorylation of the inhibitor factor κB (IF κBα). Additionally, ginseng berry extract suppresses the phosphorylation of ERK 1/2, c-Jun N-terminal kinase (JNK), and p38, reduces ROS, and increases the expression of enzymes such as glutathione peroxidase (GPxs), superoxide dismutase (SOD), and CAT. These effects are believed to be mediated by the inhibition of MAPKs signaling pathway [72].

Ginsenosides exhibit definite anti-inflammatory actions [73,74], particularly through their influence on the activation of inflammasomes. Inflammasomes are protein complexes formed by pattern-recognition receptors and other inflammatory mediators. Many ginsenosides have been shown to suppress inflammasome activation through various pathways. For instance, Rg3, Rd, Rg1, and 25-OCH3-protopanaxdiol, which are derived from *Panax* ginseng, can suppress the activation of the nucleotide-binding domain leucine-rich repeat-containing receptor, NLRP3 inflammasome. Rg3 specifically reduces the production of NO and the expression of iNOS [75], and suppresses the expression of TNF-α and the activation of NF-κB [76]. Rg1 has demonstrated the ability to suppress the activation of the nucleotide-binding domain leucine-rich repeat-containing receptor, NLRP1 inflammasome [77], and to inhibit interferon-inducible protein (AIM2) inflammasome activation in macrophages [78]. Rg1 exhibits anti-inflammatory and anti-oxidative properties, and has been shown to repair neural damages related to brain injury by inhibiting the activation of inflammasomes NLRP1 and NLRP3, or inflammasome AIM2 [76]. In addition, Rg1 demonstrates in vivo anti-inflammatory bioactivity by inhibiting interleukin receptor-associated kinase (IRAK) activation-mediated inflammatory responses. Moreover, Rg1 can inhibit the activation of the NF-κB signaling pathway [79,80]. Ginsenoside Rg5 has been shown to reduce mucin secretion, as well as to reduce the mRNA levels of the gene-encoding mucins. In an in vitro study with human lung mucoepidermoid carcinoma (NCI-H*292*) cells, the levels of MUC5AC (a mucin protein) were quantified, and it was observed that Rg5 reduced the secretion of MUC5AC, as well as the levels of MUC5AC mRNA. This study demonstrates that ginsenoside Rg5 alleviates inflammatory response by reducing mucin secretion [81]. Ginsenoside Rd has been shown to inhibit the expression of iNOS and COX-2 in later stages after ischemia [82]. Additionally, it reduces the generation of NO, production of PGE2, and activity of NF-κB [83]. Another saponin, chikusetsusaponine IVa, derived from *Panax japonicum*, a different species of ginseng, exhibits similar inhibitory actions on NLRP3 inflammasome [84,85].

Rb1 is among the most extensively studied ginsenosides. It possesses anti-inflammatory properties by suppressing IκB degradation. IκB family proteins play a crucial role in the first inhibition of NF-κB by rendering it inactive within cells. Furthermore, Rb1 prevents NLRP3 inflammasome activation and mitochondrial damage, suggesting it is an anti-inflammatory molecule [86]. Rb1 has been shown to inhibit TNF-α production in LPS-stimulated macrophages, thereby controlling inflammation and TNF-α production [87,88,89]. However, oral administration of Rb1 results in poor bioavailability, which poses challenges for its use in clinical treatment. Nevertheless, its bioavailability can be enhanced through galenic development, such as the use of various nanoformulation strategies.

Several other ginsenosides have demonstrated anti-inflammatory properties, either with similar or distinct chemical structures. For instance, Rb2 shares a very similar chemical structure to Rb1. Rb2 can also inhibit TNF-α production in cells stimulated with LPS, similar to Rb1, through the inhibition of NF-κB [90,91]. On the other hand, ginsenoside Rh1 is a considerably smaller molecule than Rb1 or Rb2, but it retains the primary structure and exhibits anti-inflammatory activity by suppressing the expression of COX-2 and iNOX [48,92].

A bacterial metabolite produced from the metabolism of ginsenoside Rh1 is ginsenoside Rh2, which also exhibits anti-inflammatory activity in microglial and astroglial cells [68,93]. Another ginsenoside derivative is ginsenoside Rp1, which has demonstrated the ability to reduce the expression of IL-1β, COX-2, and iNOS by suppressing NF-κB activity in vitro [94,95], despite being less studied, compared to other ginsenosides.

The bioactive compounds present in ginseng are widely recognized. However, ginseng also harbors other significant bioactive molecules, including a non-saponin component, known as gintonin, which has gained increasing interest. Gintonin is a complex made up of carbohydrates, proteins, and lipids, and it has been found to be divisible into six different subtypes. The principal bioactive compounds of gintonin are a series of lyso-phosphatidic acids, which act as ligands for specific membrane receptors, notably, the G-coupled protein receptors [96]. Gintonin exerts its effects through *Gq protein alpha subunit 11* (GαQ/11), phospholipase C, inositol triphosphate (IP3), and release of calcium, leading to the activation of Ca^2+^-activated Cl—channels. Moreover, gintonin has been shown to attenuate neuroinflammation associated with diseases, such as Alzheimer’s disease, by inhibiting reactive oxygen species formation and reducing cytokine production in the brain [97].

**Table 2:** Anti-inflammatory bioactivity of ginseng extracts and compounds. An increase is represented by (↑), and a decrease is represented by (↓).

Species	Molecular Group	Compound/Extract	Experimental Model	Result	Ref.
*Panax ginseng* C.A. Meyer	Ginsenoside	Rg1	ICR mice	Suppression NLRP1 inflammasome activation	[75]
	Ginsenoside	Rg1	Murine RAW 264.7 macrophage cell line	↓ IL-6	[78]
	Ginsenoside	Rg1	C57BL/6 mice	Inhibition NF-κB pathway	[79]
	Ginsenoside	Rg1	ICR mice	Inhibition NF-κB pathway ↓ iNOS ↓ COX-2	[80]
*Panax ginseng* C.A. Meyer	Ginsenoside Ginsenoside	Rg3 Rg5	BV-2 microglial cells Neuro-2a cells NCI-H292 cells	Suppression TNF-α and NF-κB ↓ MUC5AC and ↓ MUC5AC mRNA	[76,81]
	Ginsenoside	Rd	Sprague–Dawley rats	↓ iNOS ↓ COX-2	[82]
	Ginsenoside	Rd	Murine RAW 264.7 macrophage cell line	↓ iNOS ↓ COX-2	[83]
	Ginsenoside	Rb1	Sprague–Dawley rats	↑ IκB	[86]
	Ginsenoside	Rb1	Murine RAW 264.7 macrophage cell line	Suppression TNF-α	[88]
	Ginsenoside	Rb1	ICR mice	Inhibition NF-κB pathway	[87]
	Ginsenoside	Rb1 y Rb2	Murine RAW 264.7 macrophage cell line	↓ TNF-α	[90]
	Ginsenoside	Rb2, Rd, Re and Rg1	Murine N9 microglial cell line	↓ TNF-α ↓ NO	[91]
	Ginsenoside	Re and Rh1	Murine RAW 264.7 macrophage cell line	↓ iNOS ↓ COX-2	[48]
	Ginsenoside	Rh2	BV-2 microglial cells	↓ NO ↓ COX-2 ↓ TNF-α ↓ IL-1β	[93]
	Ginsenoside	Rp1	Murine RAW 264.7 macrophage cell line	↓ iNOS ↓ COX-2 ↓ IL-1β	[94]
*Panax ginseng* C.A. Meyer	Ginsenoside	Rp1	Murine RAW 264.7 macrophage cell line	↓ IL-1β ↓ iNOS ↓ COX-2 ↓ TNF-α	[95]
	-	Root water extract (saponin fraction)	Murine RAW 264.7 macrophage cell line	↓ iNOS ↓ COX-2 ↓ TNF-α	[71]
	-	Berry extract	Murine RAW 264.7 macrophage cell line	↓ iNOS ↓ COX-2 ↓ IL-1β ↓ IL-6 ↓ TNF-α	[72]
	Glycolipoprotein complex	Gintonin	SH-SY5Y Human neuroblastoma cell line	↓ ROS formation	[97]
*Panax japonicum* C.A. Meyer	Saponines	Chikusetsusaponine Iva	THP-1 human monocyte-like cells	↓ iNOS ↓ TNF-α ↓ IL-6 ↓ IL-1β	[85]
*Panax notoginseng* Burk.	Ginsenosides	Rb1	Murine RAW 264.7 macrophage cell line	↓TNF-α ↓ IL-6 ↓COX-2 ↓ IL-1β	[89]

## 5. Anti-Cancer Activity

The increase in life expectancy and modern lifestyles has led to a rise in cancer diagnoses in Western societies. The pursuit of a definitive cure for cancer appears to be a distant prospect, as different cancer types necessitate distinct treatment approaches, rendering cancer more a group of diseases than a singular entity. Hence, the discovery of compounds that either prevent cancer or aid in its treatment is of utmost significance. The findings related to the studies of the antitumor activity of ginseng are summarized in Table 3.

Various ginseng compounds, including ginsenosides [98], polysaccharides [99], and alkaloids extracted using alcohol, have exhibited different activities against distinct stages in cancer development, such as cancer cell growth, proliferation, viability, and angiogenesis processes linked to cancer. An essential factor to consider in the anti-cancer bioactivity of ginsenosides is the number of sugar moieties present. Notably, the level of anti-cancer bioactivity is inversely proportional to the quantity of sugar moieties. For instance, ginsenosides Rb1 or Rc, which have four or more sugar moieties, exhibit minimal anti-cancer bioactivity [100].

Numerous in vitro studies have investigated ginsenoside Rh1 as an anti-cancer agent [19,101]. It has been examined against various cancer types, such as human lung carcinoma (A549) or human cervix uterine adenocarcinoma (HeLa). Rh1 has been shown to exhibit antiproliferative activity on mouse fibroblast cells. The mechanism underlying this effect is the inhibition of phospholipase C, resulting in a decrease of intracellular diacylglycerol, which is an endogenous activator of protein kinase C [102]. In addition, an anti-cancer effect has been observed on a human leukemia cell line, which is attributed to the induction of apoptosis [103].

Ginsenoside Rh2 exhibits a chemical structure similar to that of dexamethasone [104]. In in vitro studies, it has been demonstrated to suppress the growth and viability of various cancer cells, induce tumor cell cycle arrest and cellular apoptosis, trigger necrosis and autophagy in cancer cells, inhibit metastasis, and suppress angiogenesis. The mechanisms associated with Rh2 have been partially elucidated and classified based on their frequency and type of action. The type of action has been divided into mechanisms related to apoptosis and autophagy, mechanisms related to cell cycle regulation, and mechanisms related to invasion and migration of cancerous cells.

Rh2 has been shown to release mitochondrial cytochrome C, reduce mitochondrial membrane potential, and activate various pathways of kinases and caspases that lead to apoptosis or autophagy processes, thereby offering protection against cancer [105,106,107]. Additionally, Rh2 has demonstrated down-regulation of cyclins, up-regulation of protein expression, and activation or regulation of different pathways leading to control cell cycle. Rh2 has also been shown to regulate the expression of proteins to prevent invasion and migration of cancerous cells. Notably, Rh2 can be used as a contributory drug to prevent drug resistance in cancer treatments. In a recent study, ginsenoside Rh2 has shown anti-cancer bioactivity against many different types of cancer, such as nasopharyngeal carcinoma, glioma, lung cancer, breast cancer, digestive system cancer, genitourinary system cancer, melanoma, or leukemia. Rh2 also helps to reduce cancer drug resistance and alleviates the side effects of classical treatments for cancer, such as chemotherapy. Depending on the type of cancer, Rh2 has been shown to intervene at one or more points in the process (cell proliferation, cell apoptosis, cell cycle, autophagy, migration, or angiogenesis) [107]. Other molecules obtained from different species of ginseng, such as 25-OH-protopanaxdiol, share this pro-apoptotic bioactivity with ginsenoside Rh2 [108].

Several ginsenosides from *Panax* species, namely, Rg1, Rg2, Rg3, and Rg5, exhibit potential anti-cancer properties. Rg1 has been found to inhibit oncogenes [109], while Rg2 increases the levels of p53 and p21, which play critical roles in regulating cellular division [110]. Rg3 is one of the most pharmacologically active ginsenosides, with a unique chemical structure that contains a hydroxyl group at C20, resulting in two different epimers, RRg3 and SRg3 [111]. Both epimers exhibit anti-angiogenic and anti-metastatic activities [112], with RRg3 showing greater potency and efficacy in inhibiting the migration and invasion of breast cancer cells in vitro [113]. Additionally, Rg3 has demonstrated both anti-proliferative and apoptotic bioactivities [114,115]. Among these ginsenosides, Rg5 exhibits the strongest anti-proliferative bioactivity [116].

In addition to the anti-cancer activities demonstrated by various ginsenosides as pure compounds, studies have also investigated the potential anti-cancer effects of different ginseng species, including the non-saponin fractions of their extracts. For instance, alkaloids and polysaccharides derived from Korean Red ginseng (*P. ginseng*) have been shown to exhibit anti-cancer activities by inhibiting the proliferation of cancer cells [117,118] and acting as an adjuvant therapy in combination with chemotherapy [103]. Moreover, the alkaloids from Korean Red ginseng have been found to exert a protective effect against chromosomal damage, promoting the repair and regrowth of radiation-damaged cells [119].

Compound K has been shown to possess anti-cancer properties through its cytotoxic effects on tumor cells [120]. It has been found to regulate tumor growth and the tumor microenvironment via various biochemical signaling pathways, and induce tumor apoptosis. These anti-cancer activities have been demonstrated in various types of cancer, including lung, bladder, colon, myeloma, and neuroblastoma [121,122]. Additionally, in lung cancer, Compound K has been observed to induce apoptosis and autophagy [123].

In *P. ginseng*, polyacetylene compounds have been extensively investigated, and they are mainly composed of panaxydol and panaxynol, which constitute approximately 90% of the total polyacetylene content. Among these compounds, panaxydol has shown the highest potency against cancer cells. Additionally, both panaxynol and panaxydol exhibit significant chemopreventive effects against oncogenesis and mutations that can lead to genetic instability. Another minor polyacetylene, panaxytriol, has been found to possess anti-proliferative effects against cancer cells [124].

A group of eight major ginsenosides (Re, Rg1, Rc, Rb1, Rb2, Rb3, Rd, and Rg3) extracted from *P. quinquefolius*, has been investigated for their anti-cancer effects [125]. Several of these ginsenosides have demonstrated anti-cancer activity, including Rg3, as we have previously discussed, as well as Rb1 [126,127], Rb3, and Rd [128]. This extract inhibited cancer cell growth and migration, as well as suppressed the invasive properties of cancer cells in a dose-dependent manner. Furthermore, it increased the expression of tumor suppressor genes p53 and p21 while down-regulating B-cell lymphoma 2 (Bcl2) and signal transducer and activator of transcription 3 (STAT3), which are crucial genes related to cancer research. Rg3, along with other ginsenosides such as Rk1 and Rg5, not present in this extract, induced cell cycle arrest in the G1 phase, resulting in the suppression of cellular proliferation, angiogenesis, and metastasis in prostate cancer [125,129,130,131].

*P. notoginseng* has ginsenoside-like molecules. Among these, 25-OH-PPD and 25-OCH3-PPD have been shown to have anti-cancer activity by inducing apoptosis and inhibiting proliferation. These compounds activate caspases and down-regulate mouse double-minute 2 homolog (MDM2), while increasing p21 gene expression [110,132,133,134,135]. Additionally, notoginsenoside R1 has demonstrated anti-cancer activity by inhibiting TNF-α and inhibiting proliferation of cancerous cells [132].

**Table 3:** Anti-cancer bioactivity of ginseng extracts and compounds. An increase is represented by (↑), and a decrease is represented by (↓).

Species	Molecular Group	Compound/Extract	Experimental Model	Result	Ref.
*Panax ginseng* C.A. Meyer	Ginsenoside	Rh1	Mouse lymphoid neoplasma cell line (P388)	Cytotoxic effect	[102]
	Ginsenoside	Rh1	Human leukemia (THP-1) cell line	↑ apoptosis	[103]
	Ginsenoside	Rh2	B16 melanoma cell line	↓ cell growth	[104]
	Ginsenoside Ginsenoside	Rh2 Rh2	Murine melanoma (B16F10) cell line, Human breast cancer line (MDA-MB-231)cell, and Hepatocyte derived cellular carcinoma (HuH-7) cell line	Anti-proliferation Anti-invasion Anti-metastasis	[106]
			Diverse cancer models	Cell cycle, autophagy, migration and angiogenesis Alleviates chemotherapy effects	[107]
*Panax ginseng* C.A. Meyer	Ginsenoside	Rg1	Osteosarcoma MG-63 cells	Oncogenes inhibition	[109]
	Ginsenoside	Rg3	Breast cancer model	Anti-proliferation	[113]
	Ginsenoside	Rg5	Hepatic Adenocarcinoma SK-HEP-1 cells	↑ p21Cip/WAF1 ↓ cyclin E ↓ CDK2 ↓ CDC25A	[116]
*Panax ginseng* C.A. Meyer	Ginsenoside	Compound K	Mouse high-metastatic melanoma B16-BL6 Human myeloid leukemia K562 Human liver cancer HepG2 Human high-metastatic lung carcinoma 95-D	↓ tumor cells	[120]
	Ginsenoside	Compound K	mouse highly metastatic melanoma (B16-BL6) Human liver cancer (HepG2) Human myeloid leukemia (K562) Human highly metastatic lung cancer (95-D)	↓ tumor cells	[121]
	Ginsenoside	Compound K	Lung cancer cells A549 and H1975	↑ autophagy ↑apoptosis	[122]
	Ginsenoside	Compound K	Bladder cancer T24 cells	↑apoptosis	[123]
	Ginsenosides	Compound K and Rb1	SKOV-3 and HEYA8 cells	↓ tumor cells survival	[126]
	Ginsenoside	Rb3 and Rd	Apc^Min/+^ mice	↓ oncogenic signaling molecules (iNOS, STAT3/pSTAT3, Src/pSrc)	[128]
	Ginsenoside	Re, Rg1, Rc, Rb1, Rb2, Rb3, Rd, Rg3, Rg5 and Rk1	Human lung cancer cells Human breast cancer cells	↑ apoptosis ↓ cell proliferation ↑ p21	[133,134,135,136]
	-	Extract	Immortalized human keratinocytes (HaCaT )cells	↑ cell viability	[118]
*Panax ginseng* C.A. Meyer	Alkaloid	-	C57BL/6 mouse spleen lymphocytes	Reparation damaged cells	[119]
	Polyacetylene compounds	Panaxydol	Murine RAW 264.7 macrophage cell line	↓ tumor cells	[124]
*Panax quinquefolius* L.	Ginsenoside	Rg3	SW-480 (Leibovitz’s L-15), HT-29 (McCoy’s 5A), and non-small cell lung (NSCLC, DMEM)	Anti-proliferation	[125]
*Panax notoginseng* Burk	Ginsenoside	Notoginsenoside R1	Sprague–Dawley rats	Cell protection	[132]

## 6. Conclusions

Different species of genus *Panax* contain numerous compounds with important properties and diverse biological activities. It is important to differentiate among species: their active compounds can differ because ginsenosides are not the only biologically active compounds, but also polysaccharides, polyacetylene compounds, and alkaloids.

Ginseng is typically consumed in combination with other medicinal plants, or enriched with vitamins and minerals, to enhance its natural properties and create a multifunctional drug.

One of the points to improve in therapy with ginsenosides is to manage a better absorption. There is an added problem as well: as ginseng is often used for its antioxidant and neuroprotective properties, it is needed to cross the blood-brain barrier. The structure of ginsenosides make this crossing difficult. Further galenic investigations are needed to improve the pharmaceutical technology which reaches the appropriate formulation to get to the nervous system.

There are still numerous compounds in ginseng that are yet to be studied, including some ginsenosides that are not present in large quantities in ginseng roots, as well as various polysaccharides and other molecules that may have significant and distinct activities, if isolated and studied individually. This presents a potential avenue for future research.

## Data Availability

Not applicable.

## Abbreviation

Abbreviation	Meaning
*Panax*	*P.*
Nitric Oxide	NO
Glucose	Glu
Arabinose in pyranose form	Arbp
Xylose	Xyl
Arabinose in furanose form	Arbf
Rhamnose	Rham
High-Performance Liquid Chromatography	HPLC
Thin Layer Chromatography/High Performance Liquid Chromatography	TLC/HPLC
Ultra High-Performance Liquid Chromatography	UHPLC
Two-Dimensional High-Performance Liquid Chromatography	HPLC 2D
Natural Killer cell	NK cell
Interleukin	IL
Tumor necrosis factor α	TNF-α
Inducible nitric oxide synthase	iNOS
Cyclooxygenase-2	COX-2
Nuclear factor κB	NF-κB
Mitogen-activated protein kinases	MAPK
Phosphatidylinositol 3-kinase	PI3K
Enzyme-Linked ImmunoSorbent Assay	ELISA
T helper type 1	Th1
Cluster of differentiation 86	CD86
Matrix metalloproteinase 1	MMP-1
Extracellular signal-regulated kinase 1/2	ERK 1/2
interferon gamma	IFN-γ
Immunoglobulin	Ig
Nuclear factor E2-related factor 2	Nrf2
Protein kinase B	PKB or Akt
Receptor activator of NF-κB ligand	RANKL
Human Embryonic Kidney cell line	HEK293 cells
Prostaglandin E2	PEG2
Toll Like receptor	TLR4
Reactive oxygen species	ROS
glutathione	GSH
Messenger RNA	mRNA
Lipopolysaccharide	LPS
c-Jun N-terminal kinase	JNK
Glutathione Peroxidase	GPxs
Superoxide dismutase	SOD
Human lung mucoepidermoid carcinoma cell line	NCI-H*292*
Catalase	CAT
nucleotide-binding domain leucine-rich repeat-containing receptor	NLRP
interferon-inducible protein	AIM2
interleukin receptor-associated kinase	IRAK
IkappaB kinase	IκB
Gq protein alpha subunit	GαQ
Inositol triphosphate	IP3
B-cell lymphoma 2	Bcl2
signal transducer and activator of transcription 3	STAT3
mouse double minute 2 homolog	MDM2
Hepatic Adenocarcinoma	SK-HEP-1 cells
Murine melanoma cell line	B16F10
Human breast cancer cell line	MDA-MB-231
Hepatocyte derived cellular carcinoma cell line	HuH-7
Immortalized human keratinocytes cell line	HaCaT
